# Evaluating a Cellular Microstructure Model Within Apoptotic Cell Death via Diffusion Magnetic Resonance and Long Diffusion Times

**DOI:** 10.1002/nbm.70148

**Published:** 2025-09-19

**Authors:** Daniel Djayakarsana, Gregory J. Czarnota, Colleen Bailey, Greg J. Stanisz

**Affiliations:** ^1^ Physical Sciences Sunnybrook Research Institute Toronto Ontario Canada; ^2^ Department of Medical Biophysics University of Toronto Toronto Ontario Canada; ^3^ Department of Radiation Oncology Sunnybrook Health Sciences Centre Toronto Ontario Canada; ^4^ Department of Radiation Oncology University of Toronto Toronto Ontario Canada

## Abstract

The growing trend for personalized cancer treatment needs a complementary system to detect treatment response more rapidly and robustly. Inducing apoptosis is still a common treatment target as the immune system can clear the apoptotic cancer cells. Many studies have demonstrated the potential of diffusion‐based MRI techniques for detecting apoptotic changes in tumors by correlating the changes of cellularity. Here we used diffusion data with long diffusion times to elucidate water exchange rates in acute myeloid leukemia cells (AML) which were acquired using stimulated echo acquisition mode (STEAM) in diffusion imaging. The two‐pool exchange model was fitted where the key parameters of interest were the intracellular fraction, $M_{I}$, intracellular exchange rate, $K_{\mathrm{IE}}$, and cell radius, *r*. Apoptosis was induced with cisplatin and significant differences were found for each of the following three parameters: $M_{I}$ decreased by ~53%, $K_{\mathrm{IE}}$ increased by ~61%, and *r* decreased by ~15%. These results highlight the potential of longer diffusion times to monitor cancer treatments that induce apoptosis.

## Graphical abstract

Here we used a two‐pool exchange model to parameterize diffusion MRI with longer diffusion times up to 800 ms. These longer diffusion times were achieved through stimulated echo acquisition mode (STEAM). We found significant differences in intracellular fraction, exchange rate, and cell radius between a control and apoptotic in vitro AML biological model.

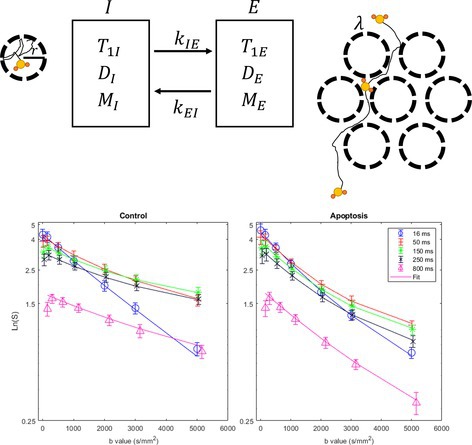

**Abbreviations**
13C: Carbon‐13
ADC: Apparent diffusion coefficient
AML: Acute myeloid leukemia
CPMG: Carr–Purcell–Meiboom–Gill
*D*
: Diffusivity
*D*_E_^app^
: Apparent extracellular diffusivity
*D*_I_^app^
: Apparent intracellular diffusivity
DTI‐STEAM‐EPI: Diffusion tensor imaging, stimulated echo acquisition mode, echo planar imaging
E: Extracellular
FBS: Fetal bovine serum
H&E: Hematoxylin and eosin
IR‐RARE: Inversion recovery, rapid acquisition with relaxation enhancement
*K*
: Exchange rate
*K*_IE_
: Intracellular water exchange rate
*M*
: Magnetization
*M*_I_
: Intracellular fraction
OGSE: Oscillating gradient spin echo
PGSE: Pulsed gradient spin echo
QUS: Quantitative ultrasound
*r*
: Radius
ROI: Region of interest
SNR: Signal to noise ratio
STEAM: Stimulated echo acquisition mode
TUNEL: Terminal deoxynucleotidyl transferase dUTP nick end labelling
US: Ultrasound
α‐MEM: Alpha‐minimum essential media
λ: Tortuosity factor

## 1 Introduction

With cancer treatments becoming more personalized with an array of different options [1, 2, 3], imaging and monitoring techniques must complement these advancements to help guide the clinician's choice [4, 5, 6]. Current solid tumor progression criteria, such as RECIST [7] and WHO [8], consider only size changes to tumors and whether new tumors are detected in remote areas [9, 10]. These size changes are far too gradual and limit the number of therapies the clinician may try on their patient; thus, we aim to explore different imaging contrasts that delve into the microstructural changes induced by therapies [11, 12, 13, 14]. The rationale behind investigating microstructural imaging markers is that microstructural changes occur prior to tumor size changes.

The microstructural changes of apoptosis are promising imaging biomarker candidates [15, 16, 17]. Many treatments such as chemotherapies and radiotherapies aim to induce apoptosis in tumors, while trying to mitigate off‐target damage. Shrinking cell sizes, increasing membrane permeability, and decreasing intracellular fraction are some characteristic effects from apoptosis caused by cellular pathways initiating the death of the cell [18].

Diffusion MRI has been used to monitor treatment response [19]. The majority of work has focused on decreases in the apparent diffusion coefficient (ADC) as cells are cleared by the immune system [20, 21, 22]. Although ADC provides information related to cell density [23], ADC encompasses all the microstructural changes into one value, losing specificity of what is occurring at the subvoxel level [24]. However, these studies report ADC rather than attempting to model cellular size and membrane permeability [20, 21, 22]. Iima et al. [25] utilize the difference in ADC resulting from different diffusion times as a predictor for malignancy. Here they find OGSE with the shorter diffusion time has an ADC of 1.28 ± 0.15 10‐3 mm^2^/s for malignant tumors and 1.99 ± 0.38 10‐3 mm^2^/s for benign tumors, while PGSE with the longer diffusion time has an ADC of 0.97 ± 0.12 10‐3 mm^2^/s for malignant tumors and 1.93 ± 0.32 10‐3 mm^2^/s for benign tumors. This time‐dependence of ADC motivates analysis methods that incorporate diffusion time information to investigate malignancy. As stimulated echo allows longer diffusion times (order of 10–100 ms) relative to OGSE and PGSE, this acquisition method is more sensitive to morphological changes caused by malignant tumors. Earlier microstructural changes related to cell size and permeability may be obtained by more advanced diffusion methods.

Other groups have demonstrated changes in kurtosis that are related to cell size and restricted diffusion [25, 26, 27, 28]. More recent in vitro diffusion work using oscillating gradient spin echo (OGSE) sequences and a parallel plane model 72 h after treatment to induce apoptosis found a decrease in the restricted size and free diffusion, as well as an increase in the restricted diffusion coefficient that reflects both membrane permeability and extracellular volume fraction [29]. Similarly, imaging microstructural parameters using limited spectrally edited diffusion (IMPULSED) was used with OGSE to determine cell sizes in tumors [16]. Information about the membrane permeability is also available at long diffusion times, but such times are challenging with conventional pulsed gradient spin echo (PGSE) sequences due to T2 signal decay [30, 31]. In this work, a stimulated echo acquisition mode (STEAM) sequence was used to examine diffusion in an in vitro model of apoptosis at a range of diffusion times up to 800 ms. A two‐pool model with exchange [32] was fit to the data to examine microstructural parameters related to cell size, intracellular and extracellular diffusivities, and transmembrane water exchange.

## 2 Methods

### 2.1 Sample Preparation

Acute myeloid leukemia cell line (AML‐5) was used, as it can be grown in suspension allowing for spherical cells, is easy to propagate, does not require trypsinization, and is well characterized for apoptosis [31, 33, 34]. There were 10 sample preparations, each sample had one control group and one apoptotic group, where every sample was performed on different days. The sample preparation followed Bailey et al. protocol [31]: AML‐5 was cultured in suspension with alpha‐minimum essential media (α‐MEM), 5% fetal bovine serum (FBS), and 1% penicillin/streptomycin in an incubator set to 37°C and 5% CO_2_. Each sample used four confluent 150 mL flasks of cells resulting in approximately $1\times 10^{9}$ cells. Apoptosis was induced with 10 μg/mL of cisplatin, a common chemotherapy drug that triggers apoptosis, for 36 h. Control cells were untreated. Each sample was then centrifuged at 2400 g to pack the cells into a pellet in a 700‐μL NMR sample tube.

The 36‐h time point was chosen based on prior studies on AML‐5 demonstrating an optimal apoptosis rate with negligible necrosis [34]. After centrifugation, the pellets remained in NMR tubes to mimic a solid tumour mass with a cell pellet density of approximately $2\times 10^{9}$ cells/mL, which matched closely with the $1\times 10^{9}$ cells in a gram of tumour [35].

### 2.2 MRI Acquisition

**FIGURE 1:**
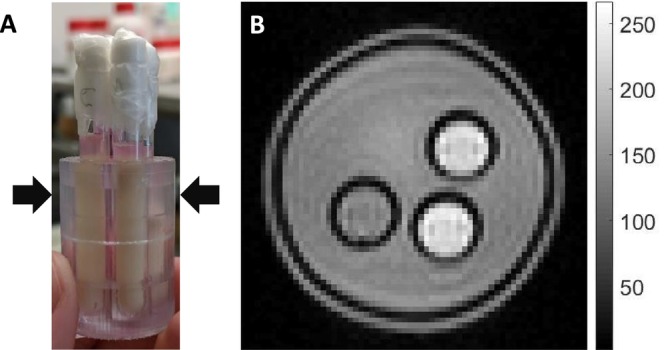
Custom 3D‐printed four‐sample holder used for MRI shown in *A*, where the arrows indicate the MRI axial image slice. The samples were control/untreated, apoptotic/cisplatin, media, and empty. A non–diffusion weighted image is shown in *B*, where the samples are blank, apoptotic, control, and media, starting at the top left going clockwise.

**FIGURE 2:**
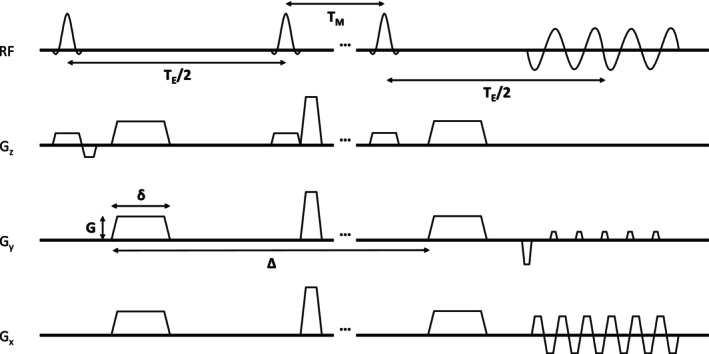
Pulse sequence diagram for diffusion‐weight stimulated echo acquisition mode with a single‐shot echo‐planar imaging readout (DW‐STEAM‐EPI). Each rf pulse has a flip‐angle of 90°. $T_{E}$ is the echo time while $T_{M}$ is the mixing time. Diffusion‐weighting is achieved with the gradient strength, G, the gradient duration, δ, and the gradient separation, Δ.

A 7 T Bruker Ascend NMR system was used to scan samples on an axial slice with a custom‐made holder at room temperature 21°C shown in Figure 1. Each scan used a control, apoptotic, and media sample. A 40/30‐mm quadrature receive and transmit coil, Micro2.5–MicWB40 with a gradient rise time of under 150 μs from 5% to 95% and a maximum gradient strength per direction of 1.5 T/m, were used to scan both samples simultaneously. Diffusion was measured with the stock Bruker DTI‐STEAM‐EPI sequence as shown in Figure 2. The spin echo crushers were omitted since they increased the diffusion weighting beyond the prescribed amounts, especially for the longer diffusion times. The diffusion gradients served as crushers, but because the non–diffusion‐weighted images did not have any diffusion gradients, they contained artefacts and were not used in the analysis. The parameters were as follows: one diffusion direction, 7 *b* values ranging from 0 to 5000 s/mm^2^, 4 signal averages, echo time TE of 35 ms, and repetition time TR of 1.5 s, varying the mixing time, TM, between 3 and 785 ms with gradient pulse length, *δ*, of 4 ms, to produce 9 different diffusion times between 16 and 800 ms (16, 30, 50, 80, 150, 200, 250, 500, 800 ms), where diffusion time was approximated as $t_{D}\sim \Delta$, Δ is the gradient separation.

For T1 relaxation measurements, inversion recovery with rapid acquisition with relaxation enhancement (IR‐RARE) was performed with TE of 9 ms, TR of 10 s, and five inversion times between 20 ms and 8 s. For T2 relaxation measurements, Carr–Purcell–Meiboom–Gill (CPMG) was performed with echo spacing of 7 ms and 180 echoes, and TR of 5 s.

### 2.3 MRI Data Analysis

Regions of interest (ROIs) were manually drawn for each NMR tube for a single slice without diffusion weighting (unweighted diffusion image) and then copied for the rest of the images. Two different diffusion methods of data analysis were used:

1. Two‐pool with exchange based on Karger equations.
2. Kurtosis

**FIGURE 3:**
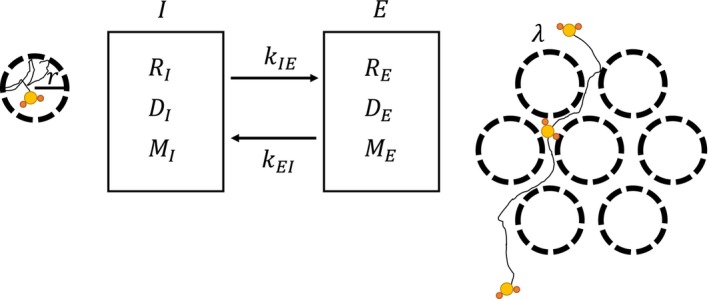
A two‐pool model of water proton longitudinal relaxation with diffusion and water exchange between the pools. The intracellular pool, I, and extracellular pool, *E*, are represented on the left and right side, respectively. The model includes a longitudinal relaxation, *T*_1_, diffusivity, *D*, and equilibrium magnetization, *M*, for both pools, with the exchange rate, *k*, between the pools. The model assumes cells are a sphere with a characteristic radius, *r*, and a tortuosity factor, λ.

A two‐pool model of water relaxation with diffusion and water exchange between the pools [32] (Figure 3) was used to fit the data. The unweighted diffusion image was ignored for fitting due to the potential artifacts from the lack of spin echo crusher.

The total magnetization in the presence of diffusion gradients was modeled with the following Bloch equations as Stanisz has done without the macromolecular pool [36, 37]:

$$\begin{array}{c} \frac{{\mathrm{dM}}_{E}}{\mathrm{dt}}=-{\left(\mathrm{\gamma g\delta}\right)}^{2}D_{E}^{\mathrm{app}}M_{E}-\frac{M_{E}}{T_{1E}}-k_{\mathrm{EI}}M_{E}+k_{\mathrm{IE}}M_{I}\\ \frac{{\mathrm{dM}}_{I}}{\mathrm{dt}}=-{\left(\mathrm{\gamma g\delta}\right)}^{2}D_{I}^{\mathrm{app}}M_{I}-\frac{M_{I}}{T_{1I}}-k_{\mathrm{IE}}M_{I}+k_{\mathrm{EI}}M_{E} \end{array}$$

where *γ* is the gyromagnetic ratio for hydrogen, *g* is the gradient strength, δ is the gradient pulse length, *M* is the magnetization, *k*_IE_ *, k*_EI_ are the exchange rate constants, *T*_1_ is the longitudinal relaxation constant, which will be referred to as just relaxation for brevity, and the subscripts *E* and *I* represent extracellular and intracellular pools, respectively. For more details on the signal equation and its derivation, see the Supplementary Materials.

Eight parameters were fitted to each dataset: *r*, *k*_IE_, *M*_I0_ (first section), *D*_Iapp_, *D*_Eapp_, *S*_0_, *T*_1I_, and *T*_1E_ *,* where *S*_*0*_ is the normalization factor capturing the proton spin density and scanner scaling. Because STEAM was used and only the mixing time was varied, only the longitudinal relaxation *R*_1_ was modeled.

Data fitting was performed by fitting the model per experiment, that is, with all the *b* values, omitting the image without diffusion weighting (*b*_0_), and all the different diffusion times, totaling 42 points per fit out of 49 data points. MATLAB's nonlinear least‐squares curve fitting (lsqcurvefit) was used to fit the data using the trust‐region‐reflective least squares algorithm to minimize the sum of squares. The errors of fitting were evaluated using the reduced χ ^2^ value. As the diffusion times measured were not short enough to sensitize to the intracellular diffusivity, the intracellular diffusivity was fixed to a constant of 1.7 μm^2^/ms. The model was tested at various fixed intracellular diffusivities around 1.7 ± 0.57 μm^2^/ms, with minimal changes to the fitting. Significance in the MRI fitting parameters between the control and treatment group was determined using a two‐sample *t* test performed in MATLAB with a *p* value less than 0.05.

The dataset acquired with stimulated echo diffusion at varying gradient separations was also fitted with a model for ADC and kurtosis. All the nonzero *b* values were fitted, which use the ADC model with an additional order seen in the equation below, where *b* is the *b* value, *D* is the ADC, and *K* is kurtosis.

$$S=S_{0}e^{-b\mathrm{ADC}+b^{2}D^{2}K/6}$$

The kurtosis of the higher gradient separation times was fitted using the time dependence of water exchange from Zhang et al. [26] shown below, where *K* is the kurtosis, $K_{0}$ is the kurtosis participating in water exchange, $\tau_{\mathrm{ex}}$ is the water exchange time, and $K_{\infty }$ is the kurtosis not participating in water exchange.

$$K\left(t\right)=K_{0}\frac{2\tau_{\mathrm{ex}}}{t}\left[1-\frac{\tau_{\mathrm{ex}}}{t}\left(1-e^{-\frac{t}{\tau_{\mathrm{ex}}}}\right)\right]+K_{\infty },\frac{1}{\tau_{\mathrm{ex}}}=k_{\mathrm{IE}}+k_{\mathrm{EI}}$$

Fitting errors were estimated by checking the correlation between each pair of parameters, shown in the Supplementary Materials. Two parameters were fixed with the other parameters remaining free and varied until

$$\chi^{2}\geq \chi_{0}^{2}\left[1+\frac{n_{P}}{N-n_{P}}F\left(n_{P},N-n_{P}\right)\right],$$

where $\chi^{2}$ is the reduced chi‐squared value from the fit with the fixed parameters, while $\chi_{0}^{2}$ is the reduced chi‐squared value with all the parameters optimized, $n_{P}$ is the number of parameters, *N* is the number of data points, and *F* is the *F* distribution function [38]. The correlations are shown in the Supplementary Materials.

### 2.4 Histology

Immediately after scanning, the samples were fixed in 10% formalin and kept for 2 weeks for histopathological evaluation. Then each sample was removed from the NMR tube and embedded in agarose gel. Once processed into a paraffin block, sections were taken along the length of the pellet and stained with hematoxylin and eosin (H&E). Terminal deoxynucleotidyl transferase dUTP nick end labeling (TUNEL) was also performed to validate apoptosis. The slides were imaged with an Axio Imager 2 (version M2, Carl Zeiss Canada Ltd., Toronto, ON) microscope with the Stereo Investigator (MBF Bioscience, Williston, BT) stereology system at 20× magnification [39] and then cells were manually counted and contoured in an area without histological artifacts using ImageJ. Three rectangular ROIs were chosen with around 80 cells per ROI for each group.

## 3 Results

**FIGURE 4:**
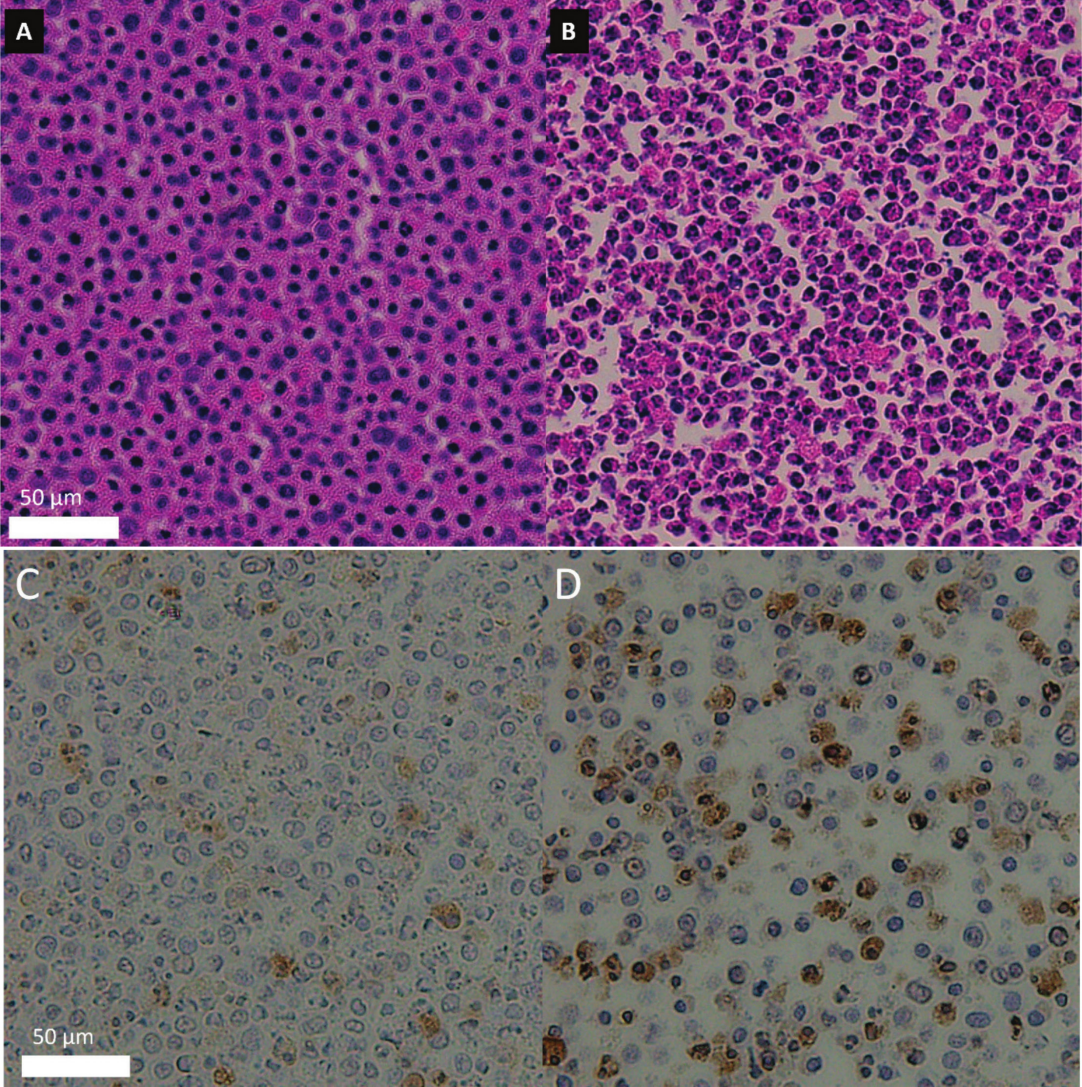
Hematoxylin and eosin‐stained histological slices of pellet post‐MRI. The left slice is the control group, A, and the right slice is the treatment group, B. The scale bar is the same for both images at 50 μm. TUNEL histological slices of pellet post‐MRI. The left slice is the control group, C, and the right slice is the treatment group, D. Brown staining indicates positive for TUNEL staining. The scale bar is the same for both images at 50 μm.

Hematoxylin and eosin histological staining of the control and treatment group post MRI is shown in Figure 4. The control group has relatively uniform sizes and a roughly circular appearance, with tighter packing relative to the treatment group. Following 36 h of cisplatin treatment, an apparent signature of apoptosis is observed: nuclear condensation and membrane blebbing are apparent with a larger distribution of cell shapes and sizes. Prior to MRI and cell pelleting, phase microscopy imaging, not shown here, also demonstrated similar morphological characteristics in the control and treatment group, such as circular and similarly sized cells in the control group and nuclear condensation and membrane blebbing in the treatment group. TUNEL staining was used as an indicator of apoptosis, where brown indicated positive staining. The control showed less staining compared to the apoptotic group, where more staining was seen around the apoptotic bodies.

T1 of the apoptotic group was 1560 ± 40 ms and the control group was 1540 ± 30 ms, while for T2 of the apoptotic group, it was 78 ± 6 ms and the control group was 68 ± 4 ms. The fitting of IR‐RARE for T1 and CPMG for T2 is not shown.

**FIGURE 5:**
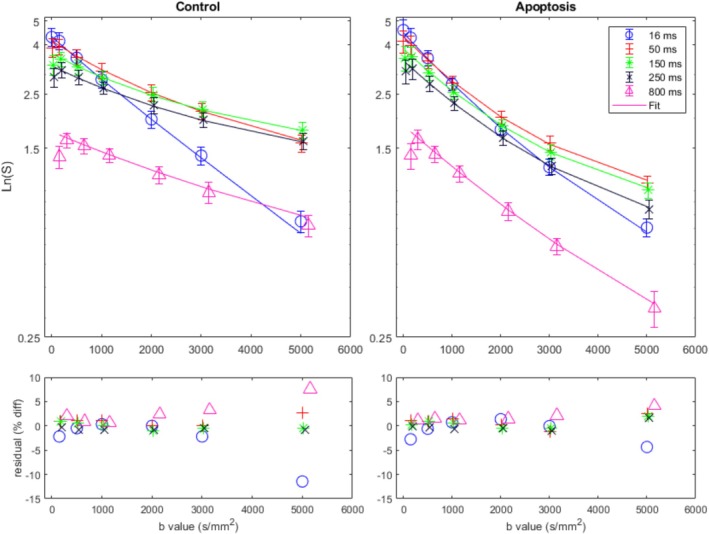
Representative two‐pool diffusion exchange model fits on an exemplary diffusion‐weighted dataset with a semilog y axis. The left column is the fit for a single control sample and the right column is the fit for a single apoptotic sample. These fits are shown in the top row with only 5 of the 9 different gradient separation times displayed for brevity. The model fits all different gradient separation times simultaneously. The error bars represent the standard deviation of the pixel variation in a single ROI. The bottom row shows the residuals in terms of percent difference.

An illustrative diffusion‐weighted dataset acquired with stimulated echo mode at varying gradient separations with the two‐pool spherical diffusion model fits is shown in Figure 5. Included in the figure are the residuals denoting the quality of the fits. The global absolute shift of the unnormalized signal downwards is seen with increasing gradient separation, and the initial slope is the highest with the shortest gradient separation of 16 ms; the slope lowers as the gradient separation increases for both groups. Divergence of the data between different gradient separations is more pronounced in the control group relative to the treatment group. The two‐pool spherical diffusion model fits all the diffusion points well, with the exception of the highest *b* values for both groups, where a larger residual is shown. The lowest *b* value point for each of the diffusion times starts lower due to artifacts from a reduced crusher necessitated by the STEAM sequence for longer diffusion times.

**FIGURE 6:**
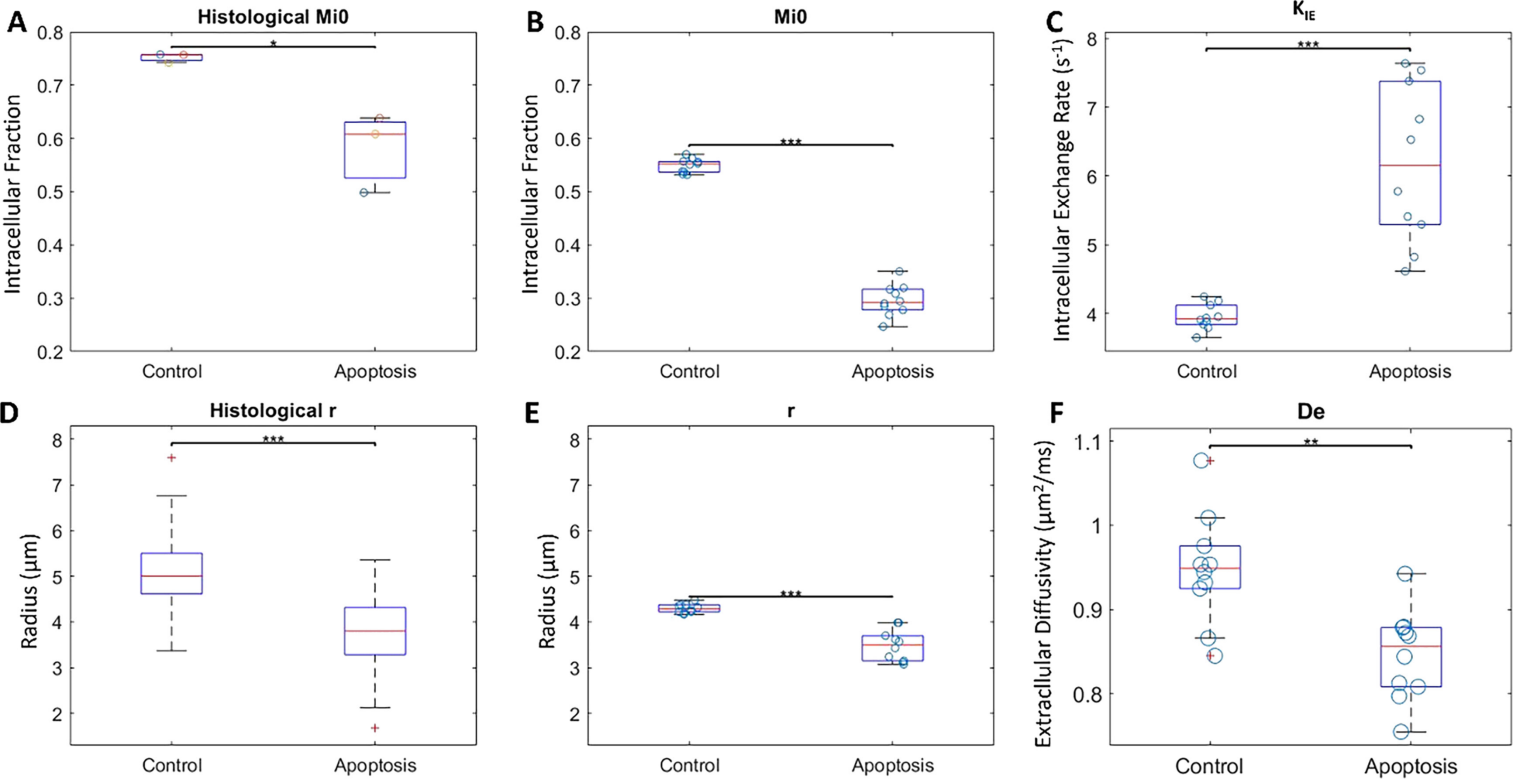
Two‐pool model fit and histological relevant parameters. Both the control and apoptotic groups are shown on each boxplot with the median marked by the red line. The left two columns, A, B, D, and E, are parameters that compare between the model fit and the histological results. The right column, C and F, only pertain to the model fit. From left to right, then top to bottom, A is the histological intracellular fraction, B is the intracellular water fraction determined by the model, C is the intracellular exchange rate, D is the histological radius, E is the characteristic radius, and F is the extracellular diffusivity. The *p* values are less than 0.05, 0.01, and 0.001 for *, ** and ***, respectively.

Relevant parameters from the model with comparison of the applicable parameters between the model and histology are shown in Figure 6 The intracellular volume fraction, $M_{I}$, and average radius, *r,* are extractable parameters from histology, although the histological slices shown in Figure 4 are two‐dimensional in its analysis causing an increase perceived intracellular fraction and larger spread in radius for the histological numbers.

There is a significant difference in all parameters between apoptotic and control cells except the intracellular relaxation as the model was unable to resolve the intracellular relaxation, where the standard deviations are averaged over the 10 samples (Figure 6). The fit quality ranged from 0.5 to 2.4 for the χ ^2^ value. For the model fitted parameters: the intracellular volume fraction was 0.32 ± 0.03 for the apoptotic cells and 0.55 ± 0.02 for the control cells, the average radius was 3.6 ± 0.2 μm for apoptotic cells and 4.2 ± 0.1 μm for control cells, the intracellular exchange rate was 6 ± 1 s^−1^ for the apoptotic cells and 4.0 ± 0.2 s^−1^ for the control cells, and the extracellular diffusivity was 0.85 ± 0.05 μm^2^/ms for the apoptotic cells and 0.95 ± 0.07 μm^2^/ms for the control cells. The intracellular longitudinal relaxation was 2 ± 3·10^−7^ s^−1^ for the apoptotic cells and 1 ± 2·10^−6^ s^−1^ for the control cells and the extracellular longitudinal relaxation was 1.7 ± 0.2 s^−1^ for the apoptotic cells and 2.9 ± 0.2 s^−1^ for the control cells. The radius from 2D histological slices was 3.3 ± 0.6 μm for apoptotic cells and 5.0 ± 0.6 μm for control cells, while the intracellular volume fraction from histology was 0.58 ± 0.07 for the apoptotic cells and 0.752 ± 0.008 for the control cells. The intracellular volume fraction, characteristic radius, and intracellular exchange rate do not have an overlap amongst their range between the control and treatment groups, while both diffusivities do have overlap between their ranges. Histological values for intracellular fraction and radius match the trend found in the two‐pool model parameters.

**FIGURE 7:**
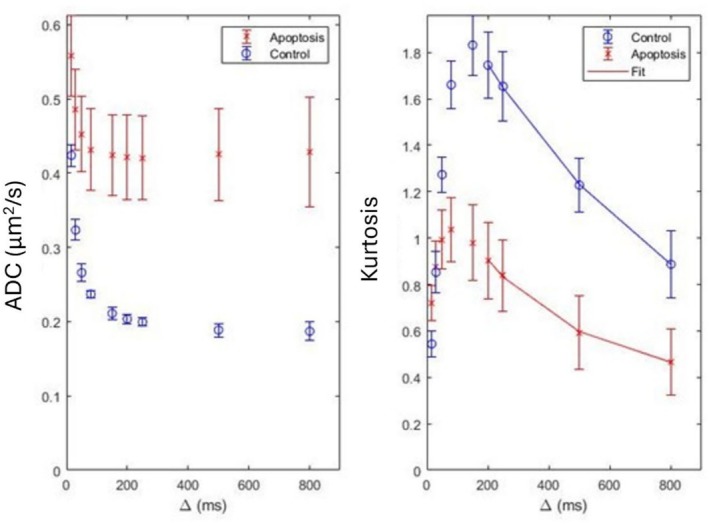
Two signal representations for diffusion MRI. The left shows the ADC dependence versus gradient separation, Δ, while the right shows kurtosis versus gradient separation. The data for both the treatment and control groups are on the same graph. Kurtosis has a model fit based on the exchange rate represented by the line fit to the four longest gradient separations (Equation 4). Nine different gradient separations were measured at 16, 30, 50, 80, 150, 200, 250, 500, and 800 ms. Error bars represent the standard deviation across the samples. *N* = 10 .

$S=S_{0}e^{-\mathrm{bD}+b^{2}D^{2}K/6}$ The dependence of ADC on the gradient separation, which is correlated to diffusion time by $t_{d}=∆-\delta /3$, is shown on Figure 7. The control group has a larger range in ADC and a longer decay time than the treatment group. Both groups' ADC values plateau at higher gradient separation. Here, the ADC in both groups had shown a significant decrease as the gradient separation times increase between 16 and 250 ms. The control group consistently has a lower ADC than the treatment group at their respective separation values.

Similarly, the kurtosis in the control group shows larger changes and peaks at a higher gradient separation time of 150 ms compared to the apoptotic group at 80 ms. After a sharp increase in kurtosis, both groups have demonstrated a more gradual decrease. The control group kurtosis is initially lower than the apoptotic group and then overtakes the apoptotic group at higher gradient separation.

For the kurtosis fitted parameters from Equation 4: the kurtosis with water exchange, *K*_*0*_, is 1.2 ± 0.1 for the apoptotic cells and 2.3 ± 0.3 for the control cells, the water exchange time, *τ*_*ex*_, is 130 ± 60 ms for the apoptotic cells and 210 ± 60 ms for the control cells, and the kurtosis without water exchange, *K*_*∞*_, is 0.1 ± 0.1 for the apoptotic cells and 0.0 ± 0.2 for the control cells. The water exchange rate, based on inverting the water exchange time, is 8 ± 4 s^−1^ for the apoptotic cells and 5 ± 1 s^−1^ for the control cells.

## 4 Discussion

We have demonstrated that microstructural apoptotic changes in vitro can be differentiated by modeling a two‐pool exchange model with diffusion MRI data. Expanding typical diffusion measurements to longer diffusion times, from ~10 to 100 ms, and higher *b* values, from ~3000 to 5000 s/mm^2^, with STEAM allowed the model to elucidate apoptotic changes of water exchange rate, cell radius, and water fraction. The two‐pool exchange model fitted multiple mixing times and *b* values with one simultaneous set of parameters.

The data demonstrated that varying the diffusion time caused drastically different signal decay curves for each diffusion time across similar *b* values. This trend across diffusion times in Figure 5 was visualized more clearly in Figure 7, depicting the different ADCs and kurtosis values. This is in agreement with previous studies that have demonstrated time‐dependent ADCs in cancer [40].

This study found a 61% increase in the water exchange rate for an AML cell line after apoptosis was induced with cisplatin. Portnoy uses a parallel plate model with PGSE and OGSE, which finds a 1700% increase in water exchange [29]. This discrepancy may be due to the parallel plates not matching the spherical nature of the cells and the lack of longer diffusion times found in STEAM necessary for the barrier‐limited exchange. Bailey uses Gd‐DTPA‐BMA in their relaxometry study to find that the exchange rate increases 380% [31]. The leakage of gadolinium into the extracellular space, especially during apoptosis with membrane integrity being compromised, could cause this larger increase in their exchange rate. Another point of contention would be that these AML samples were from different passages and phenotypic drift may be a factor [41]. Although the two‐pool exchange model did not match the non‐Gaussian compartments in the absence of exchange, the purpose of this approximation focused on characterizing the tissue parameters and comparing them to previous literature [42].

As the diffusion was non‐Gaussian, kurtosis was another avenue to parametrize the water exchange, shown in Figure 7. Kurtosis showed that the water exchange increased by 46% in the apoptotic group. This method matched well with the two‐pool exchange model as Zhang discusses the measured diffusion must also be barrier limited and coarse grained to be valid [25]. All the methods agree that apoptosis increased membrane exchange, which matches the expected trend from biology, but there is no consensus in measuring the rate [28, 29, 33, 34].

Recently, Lee shows that radii can be disentangled from water exchange using the kurtosis peak by the following equations, $t_{\text{peak}}=\sqrt{\frac{6}{5}t_{r}t_{c}},t_{c}=\frac{r^{2}}{D_{I}},t_{\mathrm{ex}}=\left(1-f\right)t_{r}$, where *f* is the intracellular water fraction and is determined by *K*_0_ with $f=\frac{K_{0}}{3+K_{0}}$ [28]. The water fraction was 0.43 with a decrease of 39% in the apoptotic group, while the radius was 9.3 μm with a decrease of 28%. The two‐pool exchange model had found the intracellular water fraction of AML was 0.55 and had decreased by 53% after apoptosis and the radius was 4.2 μm and had decreased by 15%. Vlad et al. use a Multisizer3 Coulter Counter to measure the AML particles sizes, where they find the distribution of radii is centered around 5.3 μm and a 10% decrease [43]. The explicit modeling of the spherical intracellular pool agreed better with literature and our histology measurements in the Results. Similarly, apoptosis causes cells to shrink with a decreasing water fraction as cells bleb [18]. The trends on the diffusivity (ADC) and kurtosis trends with respect to time are discussed in the Supplementary Materials [44].

There were some considerations necessary to ensure proper fitting in the analysis. In Figure 5, some of the initial data points (*b*_0_ values) were artificially lower and were not used in the fitting. These lower values were attributed to the lack of crusher gradients. Excluding these crushers was necessary to prevent unwanted diffusion weighting in stimulated echo acquisition mode as the diffusion gradients served as crushers [45]. Additionally, we used a relatively short repetition time of 1.5 s that could bias the diffusion results [46], but we compensated by adding in a dummy scan to keep imaging times lower.

On top of these scanner‐based limitations, there were three main requirements of the two‐pool exchange model. First was that the exchange must be barrier limited, where the membrane exchange rate is faster than the diffusivity [26]. The Results demonstrate that the AML model is in the barrier limited regime. Second, the diffusion time measured must be coarse‐grained, where the diffusion length is larger than the characteristic length [26]. Our model was coarse‐grained as the characteristic length, which was the cell radius of AML, was smaller than the associated diffusion length captured by longer mixing times used in STEAM‐DTI. Finally, the intracellular pool was represented by spheres with a narrow‐pulse approximation [32]. This was a limitation of the model as we were in a crossover regime due to the gradient pulse length being on the same order as the correlation time, where Fieremans demonstrates the effect of a wide pulse [47].

Diffusivity and tortuosity are shown not to be constants by Novikov and Xu [48, 49]. Here we had approximated the diffusivity and tortuosity to be constant [50], with permeability being a perturbation to the system as the longer diffusion times put the model in the tortuosity asymptote described by Latour [51]. Additionally, we tested numerous randomized initial values and found that the final fitted parameters with significant differences clustered around the same values reported in the Results, which were the water exchange rate, cell radii, intracellular water fraction, extracellular diffusivity, and extracellular longitudinal relaxivity. Here, we found the two‐pool exchange model was closely compatible with kurtosis modeling, which was expected as the basis of both was on the Kärger model and its assumptions [26, 36, 37]. The two‐pool model had statistically significant differences in the microstructural features of interest, which were the water exchange rate, intracellular water fraction, and cell radius. Thus, the potential of acquiring longer diffusion times to parameterize the two‐pool exchange model was demonstrated in an optimal in vitro model of AML as a stepping stone for future use with more complex in vivo cancer models.

In a more realistic tumour microenvironment, there are effects from the immune system, support stroma, normal tissues, necrosis, and different cancer subtypes [52]. This in vitro study did not capture any of these aspects and serves as an ideal case in the two‐pool model. Simple ADC metrics have shown promise in predicting treatment response in the clinic, so this model may prove the same, but further investigation is needed. Preclinical and clinical studies will need to be done to extend this work from in vitro to in vivo and elucidate the effect of a tumour microenvironment.

## Author Contributions

Greg Stanisz, Colleen Bailey, and Daniel Djayakarsana designed the study. Daniel Djayakarsana performed the data acquisition and analysis, wrote the draft of the manuscript, and created the figures. Greg Czarnota provided the lab space and equipment, along with the sample preparation framework of the study. Colleen Bailey and Greg Stanisz contributed to editing the drafts of the manuscript. All authors approved the final version.

## Supporting information


**Figure S1:** Correlation parameter fits for a single apoptosis group experiment from fixing two parameters and varying until the reduced chi‐squared equality is violated. The green star represents the optimized fit when all the parameters are free.
**Figure S2:** Correlation parameter fits for a single control group experiment from fixing two parameters and varying until the reduced chi‐squared equality is violated. The green star represents the optimized fit when all the parameters are free.
**Figure S3:** Two signal representations for diffusion MRI, same one in Figure 7, with inverse proportionality of gradient separation (1/Δ). The left shows the ADC, while the right shows kurtosis. The data for both the treatment and control groups are on the same graph. Nine different gradient separations were measured at 16, 30, 50, 80, 150, 200, 250, 500, and 800 ms. Error bars represent the standard deviation across the samples. N = 10.

## Data Availability

The data that support the findings of this study are available from the corresponding author upon reasonable request.
