# Supplementary material for: Evaluating a Cellular Microstructure Model Within Apoptotic Cell Death via Diffusion Magnetic Resonance and Long Diffusion Times
Source: NMR Biomed. 2025 Sep 19;38(11):e70148. doi: 10.1002/nbm.70148 (PMC12447260; doi:10.1002/nbm.70148)
Supplement: Supplementary file 1 — Figure S1: Correlation parameter fits for a single apoptosis group experiment from fixing two parameters and varying until the reduced chi‐squared equality is violated. The green star represents the optimized fit when all the parameters are free. Figure S2: Correlation parameter fits for a single control group experiment from fixing two parameters and varying until the reduced chi‐squared equality is violated. The green star represents the optimized fit when all the parameters are free. Figure S3: Two signal representations for diffusion MRI, same one in Figure 7, with inverse proportionality of gradient separation (1/Δ). The left shows the ADC, while the right shows kurtosis. The data for both the treatment and control groups are on the same graph. Nine different gradient separations were measured at 16, 30, 50, 80, 150, 200, 250, 500, and 800 ms. Error bars represent the standard deviation across the samples. N = 10. [file NBM-38-e70148-s001.docx]

## Supplementary Materials

Derivation of the two-pool model with exchange

Here are the details of the derivation of the two-pool model combining Stanisz and Price^33,37^. Here it continues with the assumption that the spins are conserved throughout the intracellular and extracellular space.

$$k_{EI}=\frac{k_{IE}M_{I0}}{M_{E0}}, M_{E0}=1-M_{I0}$$

Intracellular diffusion was modelled through restricting water in spheres^33^:

$$E\left( q,\Delta\right)= \left[ \frac{3j_{1}\left( a \right)}{a} \right]^{2}+6a^{2}\sum_{n=0}^{\infty} j_{n}^{'}\left( a \right)^{2}\sum_{m=0}^{\infty} \frac{\left( 2n+1 \right)\alpha_{nm}^{2}}{\alpha_{nm}^{2}-n^{2}-n}e^{-\alpha_{nm}^{2}\xi}\frac{1}{\left[ \alpha_{nm}^{2}-a^{2} \right]^{2}},$$

$$D_{Iapp}=-\frac{ln(E(q,\Delta))}{b}$$

where $\xi=D_{I}\Delta/r^{2},a=qr,\alpha_{nm}$ is the *m*th nonzero root of the derivative of the spherical Bessel function of the first kind. Only 200 *n* and *m* terms were used as they were sufficient to reach convergence of the aforementioned equation.

Extracellular diffusion is modelled through hindered water around the spheres^53^:

$$\left( \frac{1}{\lambda} \right)^{2}=\left( 1-f \right)^{\frac{1}{2}}, D_{E}=D_{Eapp}\lambda^{2}$$

The solution to these equations was separated into three sections: the first section prior to the diffusion pulse, the second section between the diffusion pulses, and the final section after the second diffusion pulse. The derivation was similar to Stanisz et. al^37^ in analysis of blood samples with only two pools as the macromolecular pool was omitted instead of three pools: in analysis of blood samples with only two pools as the macromolecular pool was omitted instead of three pools:

$$\frac{{dM}_{E}}{dt}=\frac{\left( A_{I}-A_{E}+Q \right)M_{E0}+2k_{IE}M_{I0}}{2Q}e^{-C_{1}t}+\frac{\left( A_{E}-A_{I}+Q \right)M_{E0}-2k_{IE}M_{I0}}{2Q}e^{-C_{2}t}$$

$$\frac{{dM}_{I}}{dt}=\frac{\left( A_{E}-A_{I}+Q \right)M_{I0}+2k_{EI}M_{E0}}{2Q}e^{-C_{1}t}+\frac{\left( A_{I}-A_{E}+Q \right)M_{I0}-2k_{EI}M_{E0}}{2Q}e^{-C_{2}t}$$

Where *A_E_*, and *A_I_*, are different for each section, *M_E0_* and *M_I0_* are the initial magnetization at the beginning of their respective section and *t* is the time at the end of their respective section.

$$C_{1}=\frac{1}{2}\left( A_{E}+A_{I}-Q \right), C_{2}=\frac{1}{2}\left( A_{E}+A_{I}-Q \right), Q=\sqrt{{(A_{E}-A_{I})}^{2}+4k_{IE}k_{EI}}$$

For the first and final section, where no diffusion weighting occurs:

$$A_{E}=R_{E}+k_{EI}, A_{I}=R_{I}+k_{IE}$$

For the second section, where diffusion weighing occurs:

$$A_{E}=R_{E}+k_{EI}+{(\gamma g\delta)}^{2}D_{Eapp}, A_{I}=R_{I}+k_{IE}+{(\gamma g\delta)}^{2}D_{Iapp}$$

$$E\left( q,\Delta\right)= \left[ \frac{3j_{1}\left( a \right)}{a} \right]^{2}+6a^{2}\sum_{n=0}^{\infty} j_{n}^{'}\left( a \right)^{2}\sum_{m=0}^{\infty} \frac{\left( 2n+1 \right)\alpha_{nm}^{2}}{\alpha_{nm}^{2}-n^{2}-n}e^{-\alpha_{nm}^{2}\xi}\frac{1}{\left[ \alpha_{nm}^{2}-a^{2} \right]^{2}},$$

$$D_{Iapp}=-\frac{ln(E(q,\Delta))}{b}\left( \frac{1}{\lambda} \right)^{2}=\left( 1-f \right)^{\frac{1}{2}}, D_{E}=D_{Eapp}\lambda^{2}$$

Errors and parameter correlations

The error fit correlations shown here were for a single experiment where the first group is for the apoptotic cohort while the second group of correlations are for the control cohort.


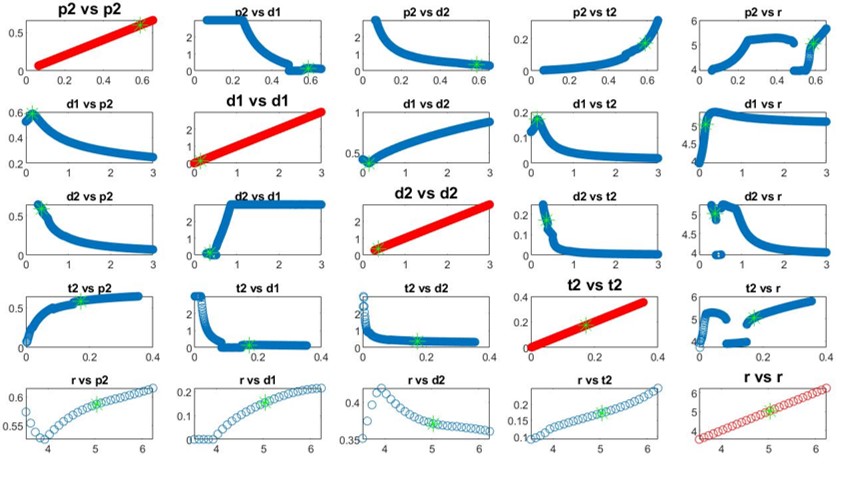


FIGURE S-1. Correlation parameter fits for a single apoptosis group experiment from fixing two parameters and varying until the reduced chi-squared equality is violated. The green star represents the optimized fit when all the parameters are free.


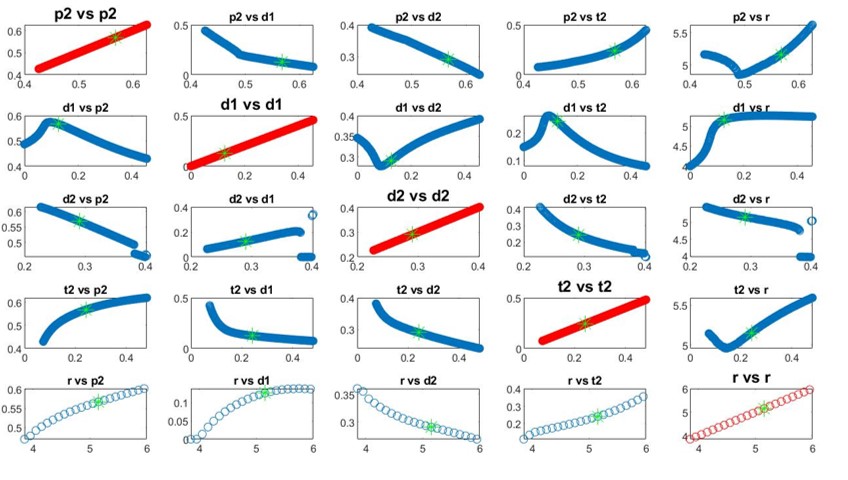


FIGURE S-2. Correlation parameter fits for a single control group experiment from fixing two parameters and varying until the reduced chi-squared equality is violated. The green star represents the optimized fit when all the parameters are free.

Diffusivity and kurtosis’ time dependence relationship


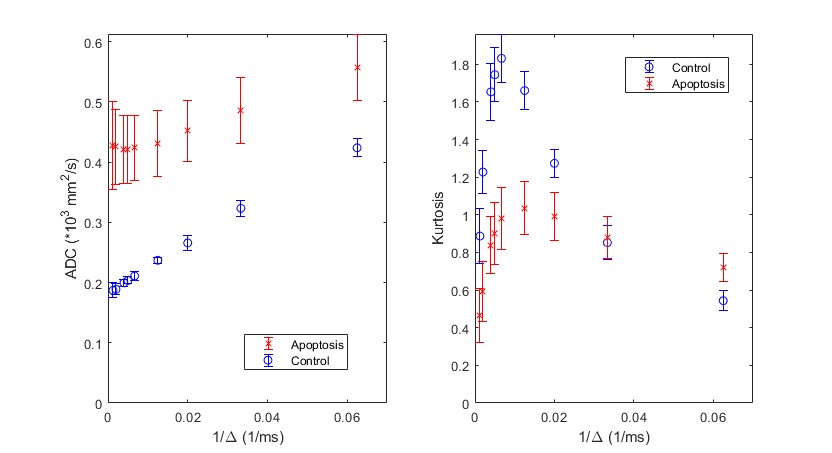
FIGURE S-3. Two signal representations for diffusion MRI, same one in Fig. 7, with inverse proportionality of gradient separation (1/Δ). The left shows the ADC, while the right shows kurtosis. The data for both the treatment and control groups are on the same graph. Nine different gradient separations were measured at 16, 30, 50, 80, 150, 200, 250, 500 and 800 ms. Error bars represent the standard deviation across the samples. N=10

With the inverse proportionality plot, the long-time limit of kurtosis was asymptotic to $K_{\infty}$ as 1/Δ approaches 0, which matched Fieremans’ theory^27^. Novikov looks at the time dependence of diffusion, where he finds the relationship to be a power-law dependent on the structure and dimensionality of the system^54^. Here we had seen potentially a 1/t relationship as 1/t approached zero. The apoptotic group had a larger error that could result in a higher value deviating from a 1/t relationship. As the system was three dimensional, this would mean the structural dimensionality was one, similar to a system of random rods with extended disorder. This was at odds with the diffusion length as it had exceeded the size of the cells, so the disorder should have become short-ranged. The closer system of tightly packed spheres would have a relationship of 1/t^2^, where permeability would have affected the relationship. Overall, the longer diffusion time trends of diffusivity and kurtosis (1/t approaches 0) had some implications from theories by Novikov and Fieremans^27,54^, respectively.

## References

1. Kato S. The Era of Personalized Cancer Treatments. *Juntendo Med J*. 2016;62(1):2-6. doi:10.14789/jmj.62.2

2. Calvaruso M, Pucci G, Alberghina C, Minafra L. Radiation Therapy Personalization in Cancer Treatment: Strategies and Perspectives. *Int J Mol Sci*. 2025;26(13):6375. doi:10.3390/ijms26136375

3. Singh D, Dhiman VK, Pandey M, et al. Personalized medicine: An alternative for cancer treatment. *Cancer Treat Res Commun*. 2024;42:100860. doi:10.1016/j.ctarc.2024.100860

4. van Houdt PJ, Saeed H, Thorwarth D, et al. Integration of quantitative imaging biomarkers in clinical trials for MR-guided radiotherapy: Conceptual guidance for multicentre studies from the MR-Linac Consortium Imaging Biomarker Working Group. *Eur J Cancer*. 2021;153:64-71. doi:10.1016/j.ejca.2021.04.041

5. Abramson RG, Arlinghaus L, Dula A, et al. MRI Biomarkers in Oncology Clinical Trials. *Magn Reson Imaging Clin N Am*. 2016;24(1):11-29. doi:10.1016/j.mric.2015.08.002

6. Soeterik TFW, Wu X, Van den Bergh RCN, et al. Personalised Prostate Cancer Diagnosis: Evaluating Biomarker-based Approaches to Reduce Unnecessary Magnetic Resonance Imaging and Biopsy Procedures. *Eur Urol Open Sci*. 2025;75:106-119. doi:10.1016/j.euros.2025.03.006

7. Eisenhauer EA, Therasse P, Bogaerts J, et al. New response evaluation criteria in solid tumours: Revised RECIST guideline (version 1.1). *Eur J Cancer*. 2009;45(2):228-247. doi:10.1016/j.ejca.2008.10.026

8. Therasse P, Arbuck SG, Eisenhauer EA, et al. New Guidelines to Evaluate the Response to Treatment in Solid Tumors. *JNCI J Natl Cancer Inst*. 2000;92(3):205-216. doi:10.1093/jnci/92.3.205

9. Lupo JM, Villanueva-Meyer JE. The Relationship Between Biological and Imaging Characteristics in Enhancing and Nonenhancing Glioma. In: Pope WB, ed. *Glioma Imaging*. Springer International Publishing; 2020:31-48. doi:10.1007/978-3-030-27359-0_3

10. Lawrence LSP, Chan RW, Chen H, et al. Diffusion-weighted imaging on an MRI-linear accelerator to identify adversely prognostic tumour regions in glioblastoma during chemoradiation. *Radiother Oncol*. 2023;188. doi:10.1016/j.radonc.2023.109873

11. Molendowska M, Palombo M, Foley KG, et al. Diffusion MRI in prostate cancer with ultra-strong whole-body gradients. *NMR Biomed*. 2024;37(12):e5229. doi:10.1002/nbm.5229

12. Tang L, Zhou XJ. Diffusion MRI of cancer: From low to high b-values. *J Magn Reson Imaging*. 2019;49(1):23-40. doi:10.1002/jmri.26293

13. Curvo-Semedo L, Lambregts DMJ, Maas M, Beets GL, Caseiro-Alves F, Beets-Tan RGH. Diffusion-weighted MRI in rectal cancer: Apparent diffusion coefficient as a potential noninvasive marker of tumor aggressiveness. *J Magn Reson Imaging*. 2012;35(6):1365-1371. doi:10.1002/jmri.23589

14. Lee HS, Kim SH, Kang BJ, Baek JE, Song BJ. Perfusion Parameters in Dynamic Contrast-enhanced MRI and Apparent Diffusion Coefficient Value in Diffusion-weighted MRI:: Association with Prognostic Factors in Breast Cancer. *Acad Radiol*. 2016;23(4):446-456. doi:10.1016/j.acra.2015.12.011

15. Jiang X, McKinley ET, Xie J, Gore JC, Xu J. Detection of Treatment Response in Triple-Negative Breast Tumors to Paclitaxel Using MRI Cell Size Imaging. *J Magn Reson Imaging*. 2024;59(2):575-584. doi:10.1002/jmri.28774

16. Xu J, Jiang X, Li H, et al. Magnetic resonance imaging of mean cell size in human breast tumors. *Magn Reson Med*. 2020;83(6):2002-2014. doi:10.1002/mrm.28056

17. McHugh DJ, Hubbard Cristinacce PL, Naish JH, Parker GJM. Towards a ‘resolution limit’ for DW-MRI tumor microstructural models: A simulation study investigating the feasibility of distinguishing between microstructural changes. *Magn Reson Med*. 2019;81(4):2288-2301. doi:10.1002/mrm.27551

18. Saraste A, Pulkki K. Morphologic and biochemical hallmarks of apoptosis. *Cardiovasc Res*. 2000;45(3):528-537. doi:10.1016/S0008-6363(99)00384-3

19. Bains LJ, Zweifel M, Thoeny HC. Therapy response with diffusion MRI: an update. *Cancer Imaging*. 2012;12(2):395-402. doi:10.1102/1470-7330.2012.9047

20. Weller A, Papoutsaki MV, Waterton JC, et al. Diffusion-weighted (DW) MRI in lung cancers: ADC test-retest repeatability. *Eur Radiol*. 2017;27(11):4552-4562. doi:10.1007/s00330-017-4828-6

21. Palmucci S, Piccoli M, Piana S, et al. Diffusion MRI for rectal cancer staging: ADC measurements before and after ultrasonographic gel lumen distension. *Eur J Radiol*. 2017;86:119-126. doi:10.1016/j.ejrad.2016.11.017

22. Pepe P, D’Urso D, Garufi A, et al. Multiparametric MRI Apparent Diffusion Coefficient (ADC) Accuracy in Diagnosing Clinically Significant Prostate Cancer. *In Vivo*. 2017;31(3):415-418. doi:10.21873/invivo.11075

23. Correlation of diffusion‐weighted magnetic resonance data with cellularity in prostate cancer. Accessed August 20, 2025. https://bjui-journals-onlinelibrary-wiley-com.myaccess.library.utoronto.ca/doi/epdf/10.1111/j.1464-410X.2008.08130.x

24. Karchevsky M, Babb JS, Schweitzer ME. Can diffusion-weighted imaging be used to differentiate benign from pathologic fractures? A meta-analysis. *Skeletal Radiol*. 2008;37(9):791-795. doi:10.1007/s00256-008-0503-y

25. Iima M, Yamamoto A, Kataoka M, et al. Time-dependent diffusion MRI to distinguish malignant from benign head and neck tumors. *J Magn Reson Imaging*. 2019;50(1):88-95. doi:10.1002/jmri.26578

26. Zhang J, Lemberskiy G, Moy L, Fieremans E, Novikov DS, Kim SG. Measurement of cellular-interstitial water exchange time in tumors based on diffusion-time-dependent diffusional kurtosis imaging. *NMR Biomed*. 2021;34(6):e4496. doi:10.1002/nbm.4496

27. Fieremans E, Novikov DS, Jensen JH, Helpern JA. Monte Carlo study of a two-compartment exchange model of diffusion. *NMR Biomed*. 2010;23(7):711-724. doi:10.1002/nbm.1577

28. Lee HH, Papaioannou A, Novikov DS, Fieremans E. In vivo observation and biophysical interpretation of time-dependent diffusion in human cortical gray matter. *NeuroImage*. 2020;222:117054. doi:10.1016/j.neuroimage.2020.117054

29. Lee HH, Novikov DS, Fieremans E, Huang SY. Revealing membrane integrity and cell size from diffusion kurtosis time dependence. *Magn Reson Med*. 2025;93(3):1329-1347. doi:10.1002/mrm.30335

30. Portnoy S, Fichtner ND, Dziegielewski C, Stanisz MP, Stanisz GJ. In vitro detection of apoptosis using oscillating and pulsed gradient diffusion magnetic resonance imaging. *NMR Biomed*. 2014;27(4):371-380. doi:10.1002/nbm.3070

31. Sanvito F, Raymond C, Cho NS, et al. Simultaneous quantification of perfusion, permeability, and leakage effects in brain gliomas using dynamic spin-and-gradient-echo echoplanar imaging MRI. *Eur Radiol*. 2023;34(5):3087-3101. doi:10.1007/s00330-023-10215-z

32. Bailey C, Giles A, Czarnota GJ, Stanisz GJ. Detection of apoptotic cell death in vitro in the presence of Gd-DTPA-BMA. *Magn Reson Med*. 2009;62(1):46-55. doi:10.1002/mrm.21972

33. Price WS, Barzykin AV, Hayamizu K, Tachiya M. A Model for Diffusive Transport through a Spherical Interface Probed by Pulsed-Field Gradient NMR. *Biophys J*. 1998;74(5):2259-2271. doi:10.1016/S0006-3495(98)77935-4

34. Czarnota GJ, Kolios MC, Abraham J, et al. Ultrasound imaging of apoptosis: high-resolution non-invasive monitoring of programmed cell death in vitro, in situ and in vivo. *Br J Cancer*. 1999;81(3):520-527. doi:10.1038/sj.bjc.6690724

35. Bailey C, Desmond KL, Czarnota GJ, Stanisz GJ. Quantitative magnetization transfer studies of apoptotic cell death. *Magn Reson Med*. 2011;66(1):264-269. doi:10.1002/mrm.22820

36. Del Monte U. Does the cell number 109 still really fit one gram of tumor tissue? *Cell Cycle*. 2009;8(3):505-506. doi:10.4161/cc.8.3.7608

37. Stanisz GJ, Li JG, Wright GA, Henkelman RM. Water dynamics in human blood via combined measurements of T2 relaxation and diffusion in the presence of gadolinium. *Magn Reson Med*. 1998;39(2):223-233. doi:10.1002/mrm.1910390209

38. Li JG, Stanisz GJ, Henkelman RM. Integrated analysis of diffusion and relaxation of water in blood. *Magn Reson Med*. 1998;40(1):79-88. doi:10.1002/mrm.1910400112

39. Bevington P, Robinson DK. *Data Reduction and Error Analysis for the Physical Sciences*. McGraw-Hill Education; 2003. https://books.google.ca/books?id=0poQAQAAIAAJ

40. Lam WW, Oakden W, Murray L, et al. Differentiation of Normal and Radioresistant Prostate Cancer Xenografts Using Magnetization Transfer-Prepared MRI. *Sci Rep*. 2018;8(1):10447. doi:10.1038/s41598-018-28731-0

41. He Z, Wilson A, Rich F, et al. Chromosomal instability and its effect on cell lines. *Cancer Rep*. 2023;6(6):e1822. doi:10.1002/cnr2.1822

42. Moutal N, Nilsson M, Topgaard D, Grebenkov D. The Kärger vs bi-exponential model: Theoretical insights and experimental validations. *J Magn Reson*. 2018;296:72-78. doi:10.1016/j.jmr.2018.08.015

43. Czarnota GJ, Kolios MC, Abraham J, et al. Ultrasound imaging of apoptosis: high-resolution non-invasive monitoring of programmed cell death in vitro, in situ and in vivo. *Br J Cancer*. 1999;81(3):520-527. doi:10.1038/sj.bjc.6690724

44. Bailey C, Desmond KL, Czarnota GJ, Stanisz GJ. Quantitative magnetization transfer studies of apoptotic cell death. *Magn Reson Med*. 2011;66(1):264-269. doi:10.1002/mrm.22820

45. Vlad RM, Saha RK, Alajez NM, Ranieri S, Czarnota GJ, Kolios MC. An Increase in Cellular Size Variance Contributes to the Increase in Ultrasound Backscatter During Cell Death. *Ultrasound Med Biol*. 2010;36(9):1546-1558. doi:10.1016/j.ultrasmedbio.2010.05.025

46. Kleinnijenhuis M, Mollink J, Lam WW, et al. Choice of reference measurements affects quantification of long diffusion time behaviour using stimulated echoes. *Magn Reson Med*. 2018;79(2):952-959. doi:10.1002/mrm.26711

47. Celik A. Effect of imaging parameters on the accuracy of apparent diffusion coefficient and optimization strategies. *Diagn Interv Radiol*. 2016;22(1):101-107. doi:10.5152/dir.2015.14440

48. Fieremans E, Burcaw LM, Lee HH, Lemberskiy G, Veraart J, Novikov DS. In vivo observation and biophysical interpretation of time-dependent diffusion in human white matter. *NeuroImage*. 2016;129:414-427. doi:10.1016/j.neuroimage.2016.01.018

49. Novikov DS, Fieremans E, Jespersen SN, Kiselev VG. Quantifying brain microstructure with diffusion MRI: Theory and parameter estimation. *NMR Biomed*. 2019;32(4):e3998. doi:10.1002/nbm.3998

50. Xu J, Xie J, Semmineh NB, Devan SP, Jiang X, Gore JC. Diffusion time dependency of extracellular diffusion. *Magn Reson Med*. 2023;89(6):2432-2440. doi:10.1002/mrm.29594

51. Latour LL, Kleinberg RL, Mitra PP, Sotak CH. Pore-Size Distributions and Tortuosity in Heterogeneous Porous Media. *J Magn Reson A*. 1995;112(1):83-91. doi:10.1006/jmra.1995.1012

52. Hanahan D, Weinberg RA. Hallmarks of Cancer: The Next Generation. *Cell*. 2011;144(5):646-674. doi:10.1016/j.cell.2011.02.013

53. Szafer A, Zhong J, Gore JC. Theoretical Model for Water Diffusion in Tissues. *Magn Reson Med*. 1995;33(5):697-712. doi:10.1002/mrm.1910330516

54. Novikov DS, Jensen JH, Helpern JA, Fieremans E. Revealing mesoscopic structural universality with diffusion. *Proc Natl Acad Sci U S A*. 2014;111(14):5088-5093. doi:10.1073/pnas.1316944111
